# Endoscopic ultrasound-guided cyanoacrylate therapy for gastric variceal bleeding secondary to sinistral portal hypertension: a retrospective case series

**DOI:** 10.3389/fmed.2026.1910217

**Published:** 2026-09-15

**Authors:** Xiaohui Ye, Lu Zhu, Yuling Zhou, Ping Li, Hongshan Wei, Lingling He

**Affiliations:** 1 Department of Gastroenterology, Beijing Ditan Hospital, Capital Medical University, Beijing, China; 2 HBV Infection, Clinical Cure and Immunology Joint Laboratory for Clinical Medicine, Capital Medical University, Beijing, China

**Keywords:** endoscopic therapy, endoscopic ultrasound, gastric variceal bleeding, gastric varices, sinistral portal hypertension

## Abstract

**Objective:**

To describe the feasibility and outcomes of endoscopic ultrasound (EUS) in the treatment of gastric variceal bleeding secondary to sinistral portal hypertension (SPH).

**Methods:**

A retrospective analysis was performed on patients with gastric variceal bleeding secondary to SPH who received EUS-guided treatment at Beijing Ditan Hospital, Capital Medical University from January 2021 to May 2025. Clinical data of the enrolled patients were retrospectively reviewed to evaluate the therapeutic effect of EUS-guided intervention for gastric varices (GVs), including variceal obliteration and recurrence, as well as early and late rebleeding events.

**Results:**

A total of four patients with SPH complicated with gastric variceal bleeding underwent EUS-guided treatment, including two males and two females, with a mean age of 35.8 years. The underlying cause was splenic vein occlusion, which was secondary to chronic pancreatitis in three patients and to a pancreatic mass in one patient. Surveillance endoscopy and contrast-enhanced computed tomography (CECT) performed at 3 months after treatment showed substantial reduction of gastric fundal varices in three patients, who subsequently completed telephone follow-up at 6 months and 1 year. No rebleeding occurred in these three patients. One female patient declined surveillance gastroscopy. 1 year post-procedure, she developed recurrent gastric variceal bleeding during the second trimester (15 weeks of gestation). No further bleeding was observed after endoscopic hemostasis. She underwent splenectomy half a year following uneventful delivery.

**Conclusion:**

EUS-guided therapy appears feasible for the management of gastric variceal bleeding secondary to SPH, particularly in patients with poor general status who are intolerant to or decline surgical treatment.

## 1 Introduction

Sinistral portal hypertension (SPH), also known as regional, segmental, splenoportal or left-sided portal hypertension, is a rare entity, accounting for less than 5% of all patients with portal hypertension (1). It typically results from splenic vein occlusion caused by acute or chronic pancreatitis and pancreatic mass, and carries a relatively low bleeding risk. Nonetheless, severe acute massive gastrointestinal hemorrhage may occur in a small subset of patients, potentially resulting in shock and fatal outcomes (2, 3).

Splenectomy is regarded as the most effective therapeutic approach. Endoscopic hemostasis serves as a bridging procedure. However, it represents a valid alternative to surgery in patients who are surgically high-risk or decline surgical intervention (4). In addition, EUS has demonstrated dual diagnostic and therapeutic values in the management of SPH. Herein, we retrospectively enrolled four patients with bleeding GVs secondary to SPH who received EUS-guided therapy. We summarized the clinical features of these patients and conducted clinical follow-up, aiming to share our clinical experience in the diagnosis and management of SPH-related hemorrhagic GVs.

## 2 Patients and methods

Between January 2021 and May 2025, a total of nine patients with SPH-related gastric variceal bleeding were evaluated at our center. Three patients underwent splenectomy, and one received conservative medical management only. Five patients refused splenectomy and interventional therapy and agreed to endoscopic treatment; one of them received conventional endoscopy instead of EUS-guided therapy due to the cost of EUS puncture needles. Ultimately, four patients underwent EUS-guided intervention and completed 1 year follow-up.

### 2.1 Inclusion criteria

The inclusion criteria were as follows:

1. Met the diagnostic criteria for SPH
2. Patients with a history of upper gastrointestinal bleeding (UGIB) who refused surgical and interventional therapy.

### 2.2 Diagnostic criteria for SPH

The diagnostic criteria include: 1) the presence of collateral vessels detected by CECT and/or gastric fundal varices found by endoscopy; 2) total or partial occlusion of the splenic vein and/or splenomegaly; 3) exclusion of idiopathic portal hypertension and liver cirrhosis (5).

### 2.3 Endoscopic procedures

All ultrasound-guided endoscopic procedures were performed by Professor Ping Li under laryngeal mask airway (LMA) anesthesia with Olympus and Pentax endoscopic ultrasound (EUS) systems.

A linear-array echoendoscope (Olympus/Pentax, Japan) visualized varices and feeding vessels. Endoscopic ultrasound -guided variceal puncture with cyanoacrylate (CYA) selective seal (EUS-PCSS) was performed to embolize the feeding vessel. The detailed procedures were as follows. Under Doppler guidance, target vessels were punctured with 22 G or 19 G fine-needle aspiration needle (Boston Scientific, hydrophilic coated). After confirming the needle position with the injection of 0.5–1.0 mL of sclerosant lauromacrogol (1%, 10 mL:100 mg; Shaanxi Tianyu Pharmaceutical Co., Ltd., Xi’an, China), a modified sandwich technique was applied, consisting of 5–10 mL sclerosant, followed by cyanoacrylate (n-butyl-2-cyanoacrylate) mixed with Lipiodol at a ratio of 1:1 according to vessel diameter, and finally 2.5 mL of normal saline. Following the procedure, EUS with Doppler imaging was used to confirm successful vascular obliteration, as evidenced by the absence of blood flow and the presence of a hyperechoic cast within the vessel.

### 2.4 Peri-procedural management

All patients received comprehensive pre-procedural assessment, including haemodynamic status, laboratory tests, contrast-enhanced abdominal CT portovenography or abdominal ultrasonography for evaluation of splenic vein and portal system. Variceal anatomy, feeding vessels, risk of ectopic embolism and general tolerance to endoscopic intervention were fully evaluated before therapy. EUS was performed only after haemodynamic stabilization was achieved in patients with active bleeding. Baseline characteristics of all patients before the procedure are summarized in Table 1.

**Table 1:** Clinical characteristics of four patients.

Parameter	Case 1	Case 2	Case 3	Case 4
Gender/Age（years）	M/44	M/36	F/27	F/36
underlying diseases	chronic pancreatitis	pancreatic tail mass	chronic pancreatitis	chronic pancreatitis
UGIB (hematemesis/ melena)	Yes	Yes	Yes	Yes
HB, (g/L)	87	99	67	90
PLT, (10^9^/L)	128	202	198	81
WBC, (10^9^/L)	3.78	2.75	3.6	3.78
ALT, (IU/L)	9.2	18.1	13.0	9.7
ALB, (g/L)	36.1	40.5	40.0	48.3
TBiL, (*μ*mol/L)	9.6	10.8	7.1	12.9
PT, (s)	14.1	10.7	14.3	11.9
INR	1.31	0.99	1.24	1.1
Hepatotropic viruses	-	-	-	-
Autoimmune liver disease antibody panel	-	-	-	-
Contrast-enhanced abdominal CT with CTPV	Normal hepatic morphology, SM, FGV, SGV, partial splenic vein occlusion,	Normal hepatic morphology, SM, FGV, SGV, splenic vein occlusion,	Normal hepatic morphology, SM, FGV, SGV, splenic vein occlusion,	Normal hepatic morphology, SM, FGV, SGV, splenic vein occlusion,
Sarin classification/RC sign	IGV1/-	IGV1/+	IGV1/+	IGV1/+
Oesophageal varices	absent	absent	absent	absent
Transfusion requirements	absent	absent	absent	absent

F, female; M, male; UGIB, upper gastrointestinal bleeding; -, negative; HGB, hemoglobin; PLT, platelet; WBC, white blood cells; ALT, alanine aminotransferase; ALB, albumin; TBil, total bilirubin; PT, prothrombin Time; INR, International Normalized Ratio; SM, splenomegaly; FGV, fundic gastric varices; SGV, short gastric veins; IGV1, Isolated gastric varices type 1; RC sign, red-color sign.

All patients were kept nil per os routinely after the procedure. A liquid diet was initiated 12 h post-procedurally if the clinical condition remained stable. Third-generation cephalosporins were given for routine antibiotic prophylaxis, with a total treatment duration no longer than 5 days. No anticoagulant therapy was performed. All patients received 24-hour routine post-procedural electrocardiographic monitoring. During hospitalization (median 5 days, range 4-7 days), Systematic physical examinations were performed daily to monitor for adverse events, with particular focus on screening for embolic-related manifestations. The assessed symptoms and signs included: fever, chest pain, chest tightness, dyspnea, tachypnea, hypoxemia, cough, hemoptysis (suggestive of pulmonary embolism); dizziness, headache, focal neurological deficits (e.g., unilateral limb weakness, slurred speech, visual-field defects) (suggestive of cerebral embolism); abdominal pain, lower-limb swelling, skin cyanosis (suggestive of systemic ectopic embolism). For patients with persistent unexplained above-mentioned symptoms, further imaging evaluations were performed, including computed tomography pulmonary angiography (CTPA), brain magnetic resonance imaging (MRI) combined with magnetic resonance angiography (MRA), abdominal/pelvic CECT, or portal-system ultrasonography.

### 2.5 Outcome measures

Technical success was defined as successful puncture and injection of cyanoacrylate (CYA) into the target vessel under EUS guidance, with observed cessation of Doppler blood-flow signal.

Complete variceal obliteration was defined as the absence of detectable blood flow within the treated varix on Doppler-EUS together with the presence of a high-echo glue cast, complete solidification of gastric varices on conventional gastroscopy, and/or absence of contrast enhancement within the varix on CECT.

Rebleeding was stratified into early (within 6 weeks post-procedure) and late (beyond 6 weeks). It was defined as hematemesis or melena accompanied by hemodynamic instability or a haemoglobin drop of >20 g/L, with endoscopic confirmation that bleeding originated from the index varix.

Persistent varix: The treated varix failed to meet the criteria for complete variceal obliteration on follow-up imaging, with persistent detectable blood flow within the target varix on Doppler-EUS, incomplete solidification on conventional gastroscopy, or positive contrast enhancement within the varix on CECT. Persistent varix indicates inadequate initial obliteration after the index procedure.

Variceal recurrence was defined as follows: complete variceal obliteration was confirmed intraoperatively or at the first post-procedural follow-up, yet flow-bearing varices re-emerged in the previously treated region on conventional gastroscopy, EUS or CECT during surveillance, including recanalization of the initially obliterated vessel or development of new varices supplying the same vascular territory.

Adverse events associated with endoscopic treatment: fever without infection, bacterial infections, systemic embolization, tissue adhesive extrusion ulcers and ulcer bleeding, and post-therapy abdominal pain. Adverse events were categorized into mild, moderate, severe, and fatal based on the American Society for Gastrointestinal Endoscopy lexicon of adverse events (6).

## 3 Results

### 3.1 Treatment and outcomes

#### 3.1.1 Case 1

A 44-year-old man with a history of chronic pancreatitis, hyperlipidemia, and hypertension presented presented to the emergency department with haematemesis. After nil-per-os status, somatostatin and proton-pump inhibitor (PPI) therapy, his vital signs stabilised and haematemesis ceased. Contrast-enhanced abdominal computed tomography (CT) with CT portal venography (CTPV) demonstrated normal hepatic morphology, splenomegaly, gastric fundal varices and short gastric veins. The main portal vein and its branches showed clear opacification without filling defects. Partial splenic vein occlusion, pancreatic tail swelling and surrounding encapsulated effusion were noted. Laboratory tests revealed hemoglobin 87 g/L and normal liver function (Table 1). Conventional gastroscopy showed isolated gastric varices. EUS was performed, which identified the short gastric vein as the feeding vessel of the fundal varices. EUS-PCSS was then performed to embolize the feeding vessel. Post-injection EUS confirmed vascular occlusion with hyperechoic cast formation (Table 2). The patient developed low-grade fever after the procedure, with a maximum temperature of 38 °C. Body temperature returned to normal approximately two days later (Table 2). Surveillance endoscopy at 3 months to assess variceal eradication showed complete solidification of the gastric varix, with no recurrent episodes of gastrointestinal bleeding (Figures 1A–E). CECT performed at the 3 months follow-up revealed no enhancing varices in the gastric fundus (Table 2). No episodes of recurrent hematemesis or melena were reported during the 1-year follow-up period.

**Table 2:** Clinical and procedural characteristics of four patients receiving EUS-guided injection therapy.

Parameter	Case 1	Case 2	Case 3	Case 4
Intervention scenario (emergent hemostasis/post-hemostasis/secondary prophylaxis)	post-hemostasis	post-hemostasis	post-hemostasis	post-hemostasis
Target vessel (varix/perforator/feeding vessel)	feeding vessel	feeding vessel	Perforator	feeding vessel
Gastrorenal shunt status	absent	absent	absent	absent
EUS-measured vessel diameter(mm)/ flow velocity(cm/s)	9.0/15	5/10	7.6/20	4.3/20
Needle gauge/ Number of punctures	22G/1	19G/1	22G/1	22G/1
Cyanoacrylate-lipiodol volume (mL)/ lauromacrogol volume (mL)	3/10	1/5	2/10	2/10
Immediate post-procedure Doppler	No detectable flow	No detectable flow	No detectable flow	No detectable flow
Peri-procedural adverse events	Mild (fever)	None	Mild (epigastric pain)	none
Total follow-up duration	1 year	1 year	1 year	1 year
Follow-up modalities at each time point	3 months: endoscopy, CECT; 6 months, 1 year: telephone follow-up	3 months: endoscopy, CECT; 6 months, 1 year: telephone follow-up	3 months: endoscopy, CECT; 6 months, 1 year: telephone follow-up	3 months, 6months: telephone follow-up; 1 year: emergency endoscopy
Rebleeding (early/late/none)	none	none	none	late
Subsequent treatment after index procedure	none	none	none	splenectomy

**Figure 1:**
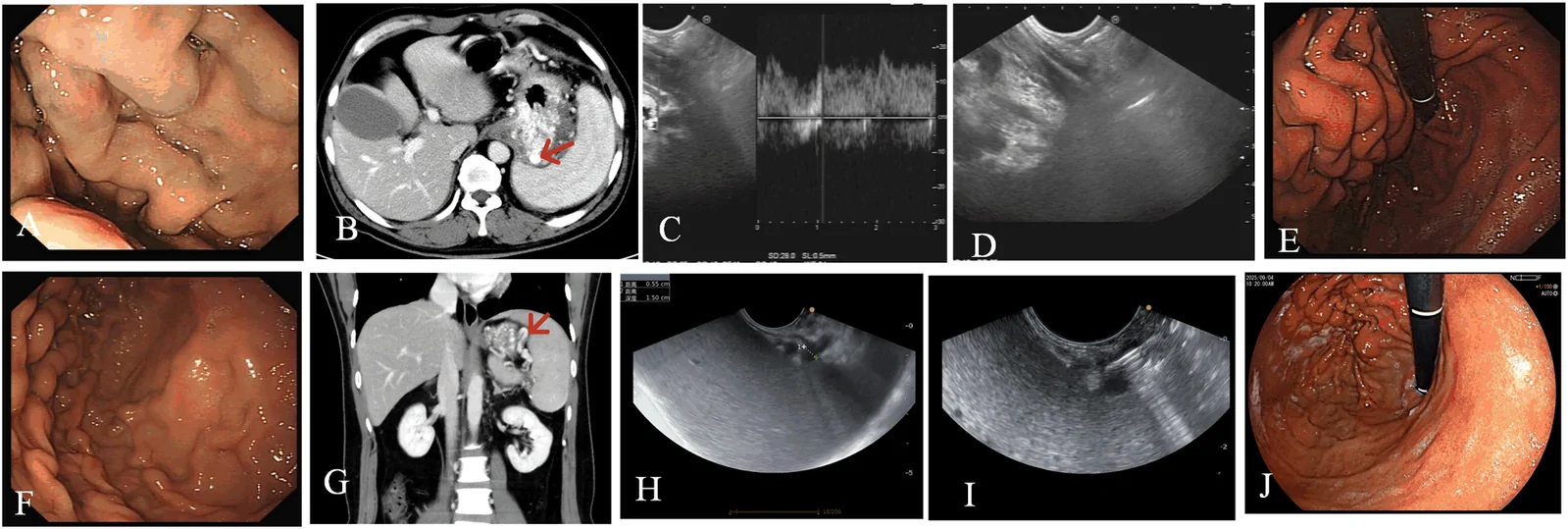
A–E correspond to case 1, and F–J to case 2; **(A)** Isolated gastric varices (pre-treatment endoscopy); **(B)** Portal venous phase CT angiography showing the short gastric veins to be the primary feeding vessel of the variceal complex (the red arrowhead), (CT obtained before endoscopic treatment); **(C)** Doppler ultrasonography showing the source vessel with peak flow velocity of 15 cm/s, (intra-procedural view); **(D)** EUS showing the source vessel trunk and some branch vessels closed by CYA, (intra-procedural view); **(E)** Follow-up conventional endoscopy at 3 months post-procedure, demonstrating substantial reduction of the fundal varices; **(F)** Isolated gastric varices (pre-treatment endoscopy); **(G)** Portal venous phase CT angiography showing the short gastric veins to be the primary feeding vessel of the variceal complex (the red arrowhead), (CT obtained before endoscopic treatment); **(H)** Doppler ultrasonography showing the feeding vessel; **(I)** Variceal puncture with CYA selective seal through a 22-gauge needle, (intra-procedural view); **(J)** Follow-up conventional endoscopy at 3 months post-procedure, demonstrating substantial reduction of the fundal varices.

#### 3.1.2 Case 2

A 36-year-old man had surgery for a pancreatic tail -mass three years ago, complicated by postoperative encapsulated fluid collection in the pancreatic tail. He was admitted due to hematemesis and melena. After nil-per-os status, somatostatin, and PPI therapy, the patient's vital signs remained stable. Contrast-enhanced abdominal CT with CTPV demonstrated normal hepatic morphology, splenomegaly gastric fundal varices and visible short gastric veins. The main portal vein and its branches showed clear opacification without filling defects. Splenic vein occlusion, pancreatic tail swelling and and linear high-density opacities at the pancreatic tail and splenic hilum were identified, consistent with post-pancreatic neoplasm resection changes. Laboratory tests revealed hemoglobin 99 g/L and normal liver function (Table 1). Conventional gastroscopy revealed gastric fundal varices with positive red color sign. EUS-PCSS was performed to treat the GVs. (Table 2). No adverse events were observed after the procedure (Table 2). Surveillance endoscopy at 3 and 6 months to assess variceal eradication showed resolution of gastric fundal varices, without recurrent episodes of gastrointestinal bleeding (Figures 1F–J). CECT performed at the 3 months follow-up revealed no enhancing varices in the gastric fundus. At telephone follow-up 1 year post-index procedure, no recurrent hematemesis or melena was reported.

#### 3.1.3 Case 3

A 27-year-old woman with chronic pancreatitis and diabetes was hospitalized due to hematemesis and melena. After nil-per-os status, somatostatin, and PPI therapy, the patient's vital signs remained stable. Contrast-enhanced abdominal CT with CTPV performed at another hospital showed normal hepatic morphology, peripancreatic tail effusion complicated with pseudocyst formation, splenic vein occlusion and gastric fundal varices. Laboratory tests revealed hemoglobin 67 g/L and normal liver function (Table 1). Conventional gastroscopy demonstrated isolated gastric fundal varices. EUS-PCSS was subsequently performed to treat the GVs (Table 2). The patient developed mild epigastric pain after the procedure, which resolved approximately one day later (Table 2). Follow-up endoscopy at 3 months showed regression of gastric fundal varices with no recurrence of gastrointestinal bleeding (Figures 2A–E). CECT revealed no enhancing varices in the gastric fundus. At telephone follow-up 1-year post-index procedure, no recurrent hematemesis or melena was reported.

**Figure 2:**
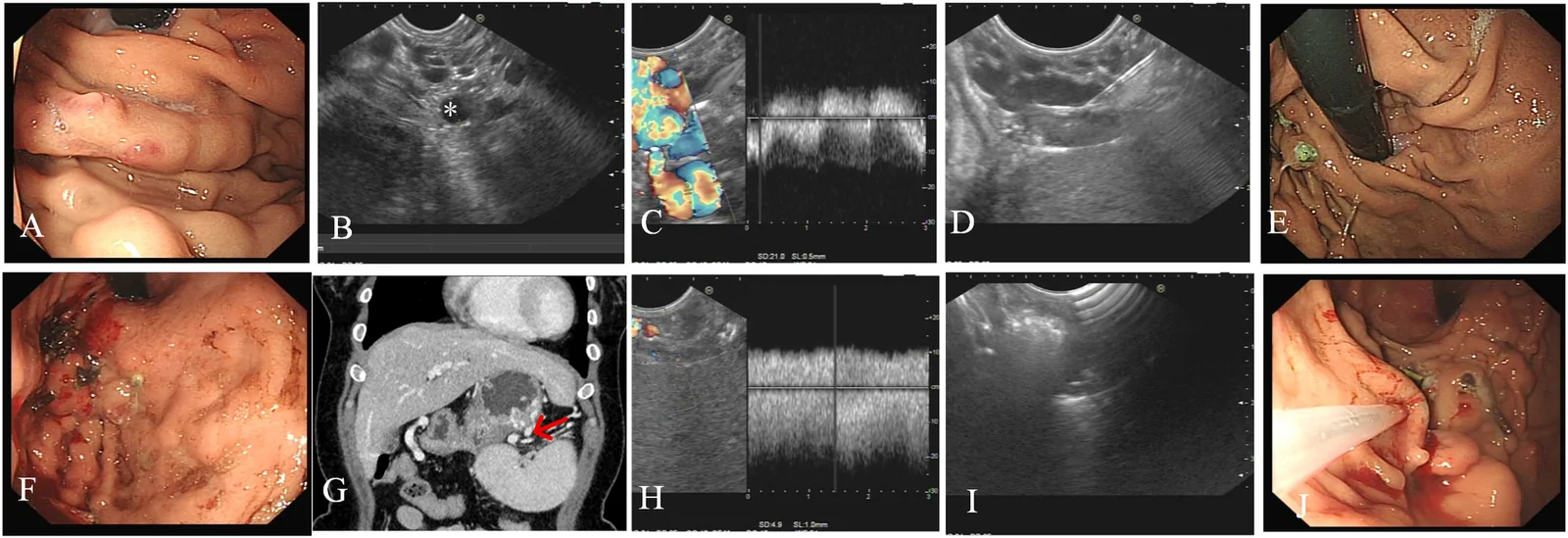
A–E correspond to case 3, and F–J to case 4; **(A)** Isolated gastric varices with positive red color sign (pre-treatment endoscopy); **(B)** Doppler ultrasonography showing perforating branch vessel (white star), (intra-procedural view); **(C)** Doppler ultrasonography showing the perforating branch vessel with peak flow velocity of 20 cm/s, (intra-procedural view); **(D)** EUS showing the perforating branch vessel closed by CYA, (intra-procedural view); **(E)** follow-up conventional endoscopy at 3 months post-procedure, demonstrating substantial reduction of the fundal varices; **(F)** Isolated gastric varices with active oozing (pre-treatment endoscopy); **(G)** Portal venous phase CT angiography showing the primary feeding vessel of the variceal complex (the red arrowhead), (CT obtained before endoscopic treatment); **(H)** Doppler ultrasonography showing the feeding vessel with peak flow velocity of 20 cm/s, (intra-procedural view); **(I)** EUS showing the feeding vessel closed by CYA, (intra-procedural view); **(J)** 1 year later, emergency endoscopic CYA injection for ruptured gastric fundal variceal bleeding;.

#### 3.1.4 Case 4

A 36-year-old woman had a history of chronic pancreatitis and diabetes. She was admitted due to hematemesis and melena. Emergency gastroscopy at another hospital revealed gastric fundal variceal bleeding, which was managed with hemostatic clip placement. After admission, the patient received somatostatin and PPI therapy, and her vital signs remained stable. Contrast-enhanced abdominal CT with CTPV showed normal hepatic morphology, splenomegaly fundal gastric varices and visible short gastric veins. The main portal vein and its branches showed clear opacification without filling defects. Splenic vein occlusion and a peripancreatic-head pseudocyst were noted. Laboratory tests revealed hemoglobin 90 g/L and normal liver function (Table 1). Conventional gastroscopy demonstrated isolated gastric fundal varices. Subsequently, EUS-PCSS was performed to treat the GVs (Table 2). No adverse events were observed after the procedure (Table 2). Telephone follow-up at 3 and 6 months revealed no recurrent gastrointestinal bleeding, yet the patient refused follow-up endoscopy. 1 year later, the patient presented with hematemesis again at 15 weeks of gestation. The patient had a strong desire to continue the pregnancy and deliver, and refused splenectomy. Therefore, emergency endoscopy and endoscopic CYA injection were performed immediately, confirming gastric fundal variceal bleeding (Figures 2F–J). After endoscopic hemostasis, no rebleeding occurred. The patient delivered a healthy full-term infant uneventfully and underwent splenectomy 6 months after delivery.

## 4 Discussion

The most common cause of SPH is acute or chronic pancreatitis, followed by pancreatic tumors and related surgeries (3, 7). In contrast to patients with cirrhotic portal hypertension, The majority of affected individuals develop isolated gastric fundal varices, with a low prevalence of concomitant esophageal varices (2). The risk of bleeding from varices is relatively low, with rates varying across studies, ranging from approximately 1.92% to 19.1% (5, 8–10). However, severe acute gastrointestinal bleeding, and even shock or death, may occur in a minority of patients. SPH remains the only curable form of portal hypertension. Accordingly, prompt hemostasis is critically important to allow time for definitive subsequent management in patients with SPH complicated by acute gastrointestinal bleeding.

However, standardized treatment guidelines for SPH are still lacking. Current main treatment approaches include surgery, interventional radiology, and endoscopic therapy. Among these, splenectomy is widely accepted as the definitive standard treatment, as it can rapidly normalize gastric fundal variceal pressure and achieve effective hemostasis in patients with refractory bleeding (11). However, the spleen represents an essential immune organ, and splenectomy may result in impaired immune function as well as overwhelming post-splenectomy infection (OPSI), which carries an incidence of approximately 0.1%–0.5% and a mortality rate of up to 50% (12). Therefore, it is crucial to appropriately select candidates for splenectomy in patients with SPH, although no definitive consensus exists thus far. Several researchers have advocated that surgical intervention is not warranted in asymptomatic patients with SPH in the absence of bleeding. Conservative treatment may be considered for those without recurrent or life-threatening hemorrhage (1, 13).

Interventional and endoscopic therapies serve as conservative treatment options for SPH. For patients who refuse surgery or have severe comorbidities, interventional and endoscopic procedures may be used as alternatives to surgical treatment (4). Partial splenic artery embolization is a commonly used interventional procedure for SPH complicated with gastrointestinal bleeding, somewhat akin to non-surgical “splenectomy”. However, a post-embolization syndrome with left upper quadrant pain and fever can be observed. The most severe complications include partial infarction of the stomach or pancreas, splenic abscess, hematoma and/or late splenic rupture (14, 15).

Endoscopy offers the advantage of identification and simultaneous treatment of bleeding varices. Moreover, endoscopy is particularly useful in the emergency setting before embolization or splenectomy, which is suitable for patients who refuse surgery or have severe comorbidities (4).

Endoscopic therapies include endoscopic band ligation, sclerotherapy, and tissue adhesive injection. For the treatment and secondary prophylaxis of acute gastric variceal bleeding, tissue adhesive injection (e.g., N-butyl cyanoacrylate) is recommended as the first-line approach (16).

A randomized controlled study demonstrated that in the control of acute gastric variceal bleeding, the CYA injection group achieved a higher hemostasis rate than the band ligation group (93.9% vs. 79.5%; *P* = 0.03) and was also superior in preventing variceal recurrence (36.0% vs. 66.0%; *P* = 0.002) (17).

Another prospective randomized study found that CYA injection was superior to endoscopic ligation in reducing the rebleeding rate in the management of gastric variceal bleeding (31% vs. 54%; *P* = 0.0005) (18).

However, endoscopic CYA injection is associated with potential complications, including systemic embolization (1.0%–4.4%), extrusion ulcer with bleeding (0.1%–10.25%), and sepsis (1.3%–3.89%) (19–21).

Owing to these limitations, the efficacy of gastric variceal treatment is inferior to that of esophageal variceal management. To minimize the above complications, researchers have suggested achieving hemostasis with the minimal necessary volume of CYA as much as possible (22, 23). Endoscopic follow-up is recommended to confirm variceal obliteration, thereby reducing the rates of rebleeding and variceal recurrence. However, it is difficult to confirm complete obliteration of varices under conventional endoscopy.

Moreover, identifying the optimal injection site under conventional endoscopic visualization can be difficult, especially in patients with ulcers or scars formed after previous tissue adhesive therapy. EUS-guided CYA injection appears to overcome the above limitations.

EUS can visualize the venous systems supplying the varices, such as the left gastric vein, posterior gastric vein, or short gastric veins (24), while also enabling identification of gastrorenal shunts (25). Even in the setting of active bleeding, EUS can accurately identify GVs and localize the feeding vessels, which is difficult to achieve with conventional endoscopy due to poor visualization (26, 27).This superior visualization under EUS guidance can translate into fewer incomplete obliterations, a lower volume of sclerosant or CYA required, and a potentially reduced risk of systemic embolization (28).

A non-concurrent cohort study of 104 patients with GVs revealed that EUS-guided tissue adhesive injection was superior to direct endoscopic tissue adhesive injection. The EUS-guided group required a smaller volume of CYA (2.0 ± 0.8 mL vs. 3.3 ± 1.3 mL; *p* < 0.001), treated more variceal columns per session (1.6 ± 0.7 vs. 1.1 ± 0.4; *p* < 0.001), and achieved lower 30-day rebleeding rate (8.8% vs. 23.7%; *p* = 0.045) and overall rebleeding rate (5.3% vs. 18.4%; *p* = 0.041). However, the incidence of adverse events was comparable between the two groups (20.3% vs. 17.5%; *p* = 0.723) (29). A network meta-analysis by *Brigida et al*. demonstrated that EUS-guided cyanoacrylate and EUS-guided coil + cyanoacrylate injection were associated with significantly lower rebleeding rates versus conventional endoscopic cyanoacrylate injection (RR = 0.39, 95% CI 0.27–0.58; RR = 0.15, 95% CI 0.03–0.90, respectively) (30).

We herein report four cases of SPH complicated with gastrointestinal bleeding treated with EUS. Under EUS guidance, perforating branches or feeding vessels were accurately identified and substantially reduced. Three patients experienced no recurrent bleeding, and follow-up endoscopy or CECT showed marked resolution of gastric fundal varices.

One patient failed to undergo timely follow-up endoscopy due to pregnancy and subsequently developed recurrent gastrointestinal bleeding during the second trimester. Emergent endoscopy revealed gastric fundal bleeding. Considering the absence of Doppler flow signals immediately after the initial EUS procedure and persistent segmental splenic vein obstruction, we hypothesized that recurrent variceal bleeding in this patient resulted from variceal recurrence. Addario et al. (31) reported that pregnancy may exacerbate bleeding events in patients with SPH. Pregnancy may elevate splanchnic blood flow, which might serve as a potential trigger for variceal recurrence.

### 4.1 Limitations

This study has several inherent limitations. First, it was a retrospective, single-center investigation with a small sample size (*n* = 4) and relatively short follow-up duration, which may limit its generalizability. Given the limited number of cases, substantial selection bias cannot be ruled out. The lack of a control group also precluded adequate verification of the definitive efficacy and safety of this technique. Second, much of the available evidence for CYA injection originates from patients with cirrhotic-related gastric varices, and these findings may not be directly generalizable to those with gastric variceal bleeding secondary to SPH. Accordingly, further randomized controlled studies are needed to verify both the efficacy and safety of CYA injection in the SPH population. Third, in consideration of the economic burden on both healthcare systems and patients, adjunctive coil embolization was not applied in any patient of this cohort. Therefore, future large-scale, prospective randomized controlled trials are warranted to comprehensively compare the efficacy, safety, and cost-effectiveness of EUS-guided cyanoacrylate monotherapy versus combined coil-cyanoacrylate therapy in patients with SPH-related gastric variceal bleeding.

## 5 Conclusion

EUS-guided cyanoacrylate injection appears feasible for the management of gastric variceal bleeding secondary to sinistral portal hypertension (SPH). In particular, it is a viable option for patients with severe comorbidities who cannot tolerate or refuse surgery.

## Data Availability

The raw data supporting the conclusions of this article will be made available by the authors, without undue reservation.
